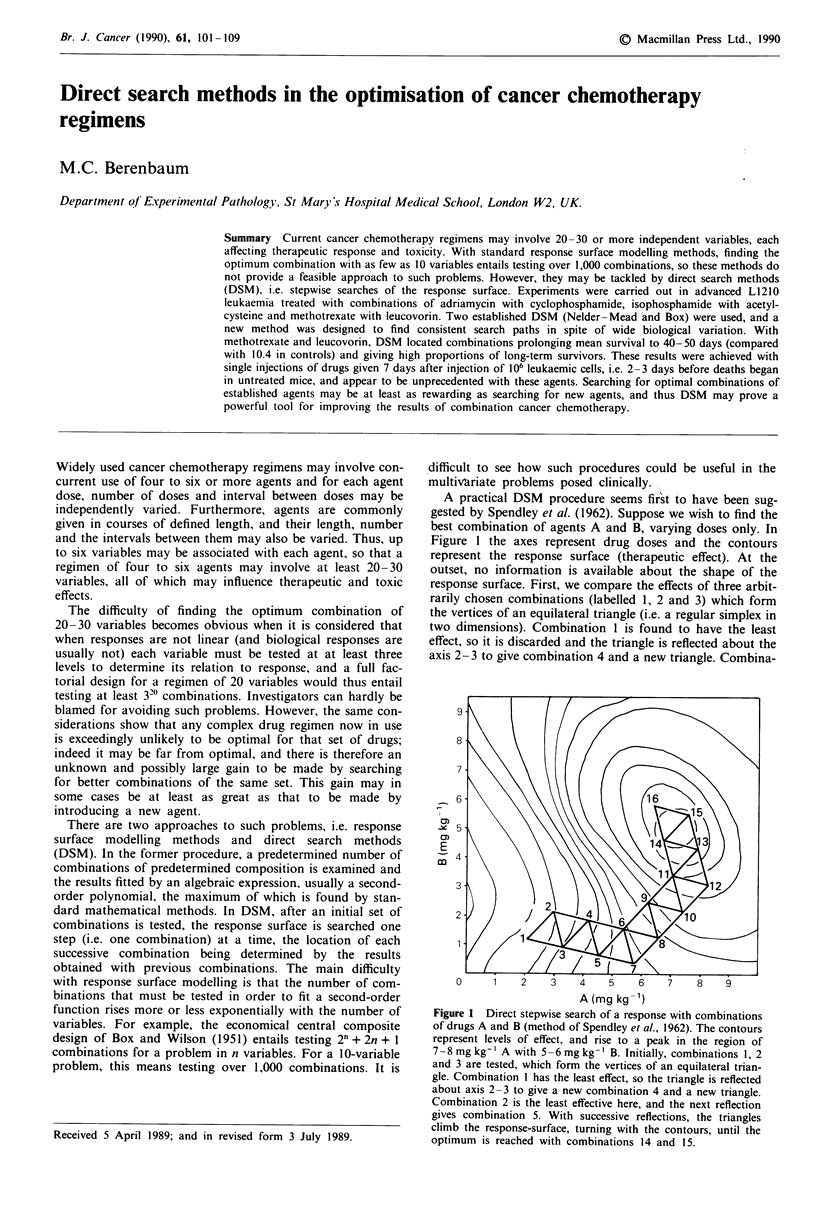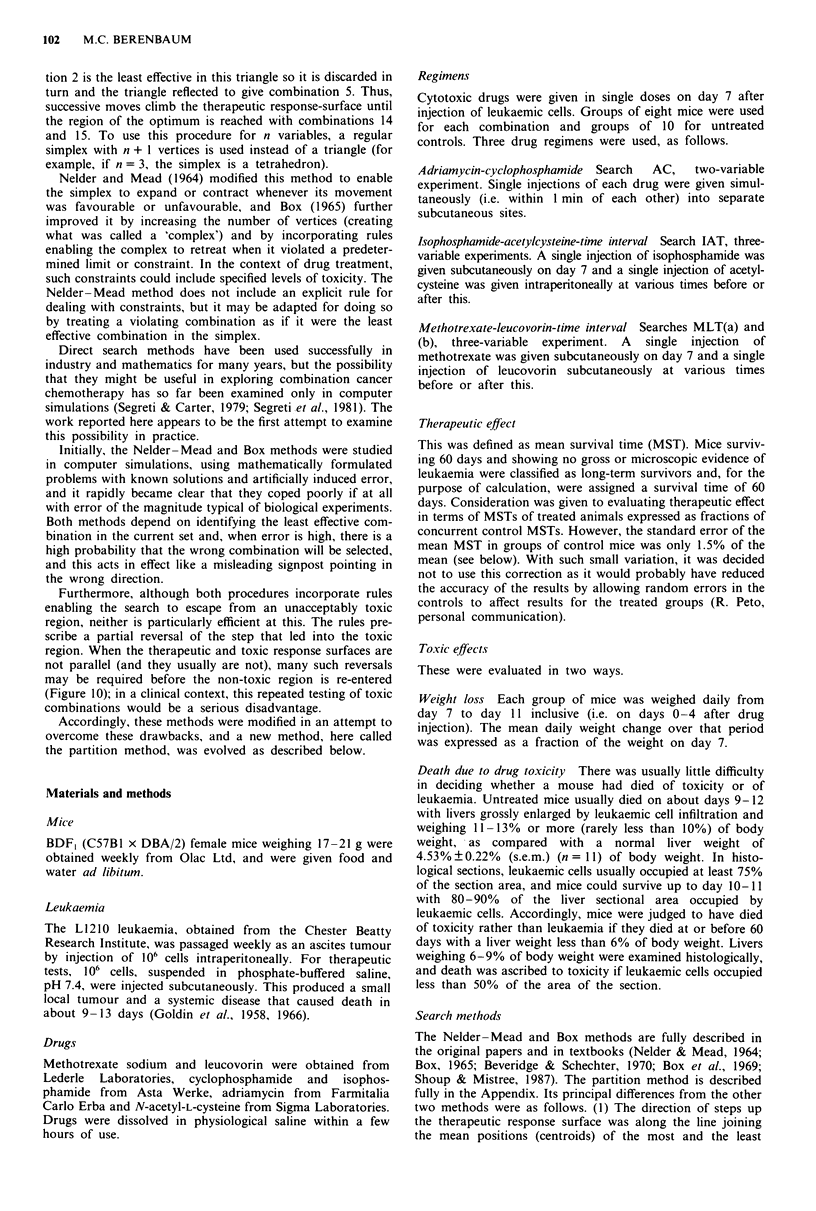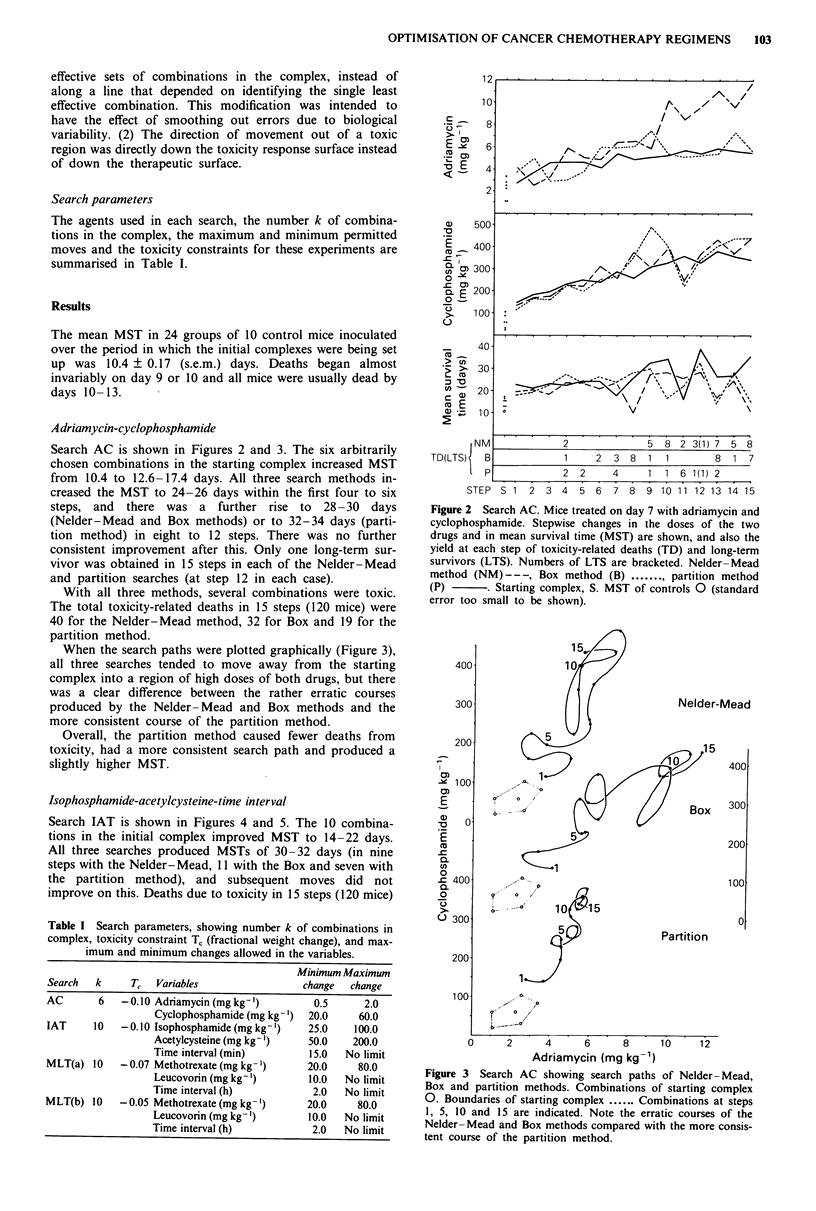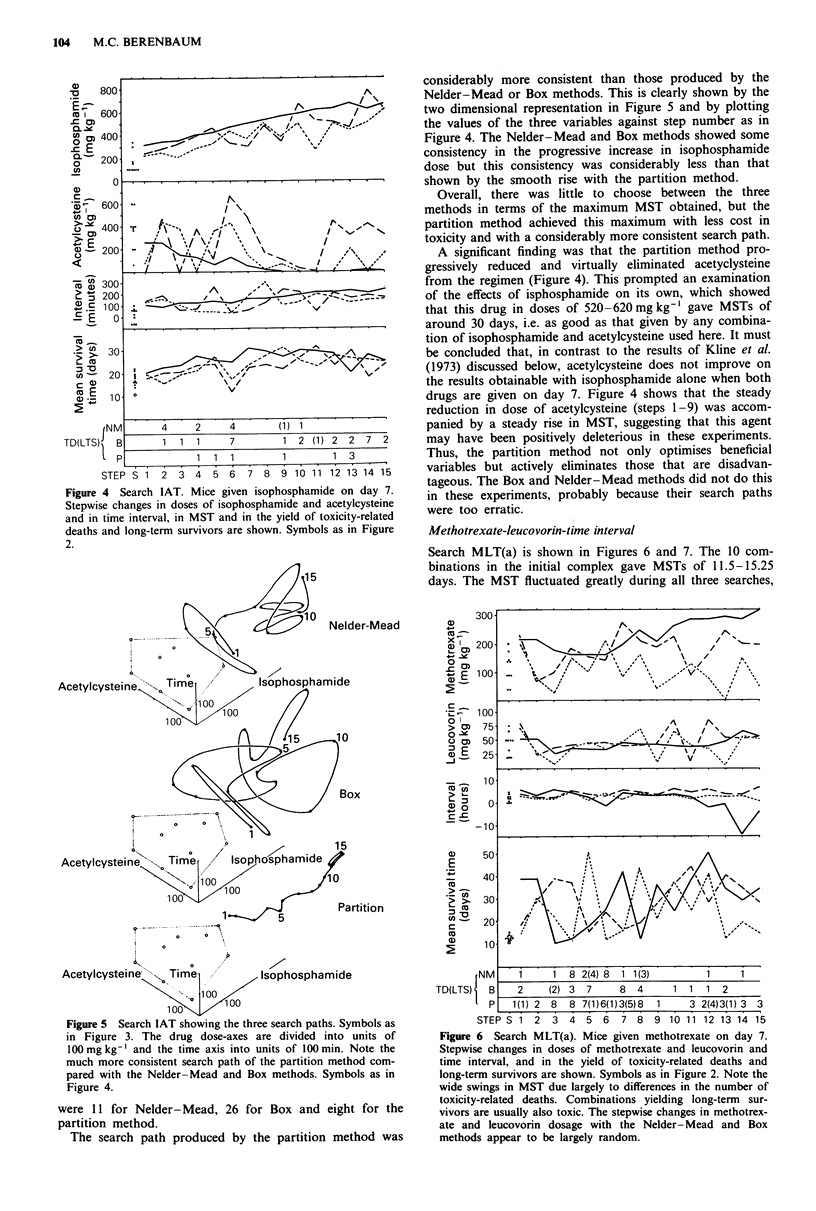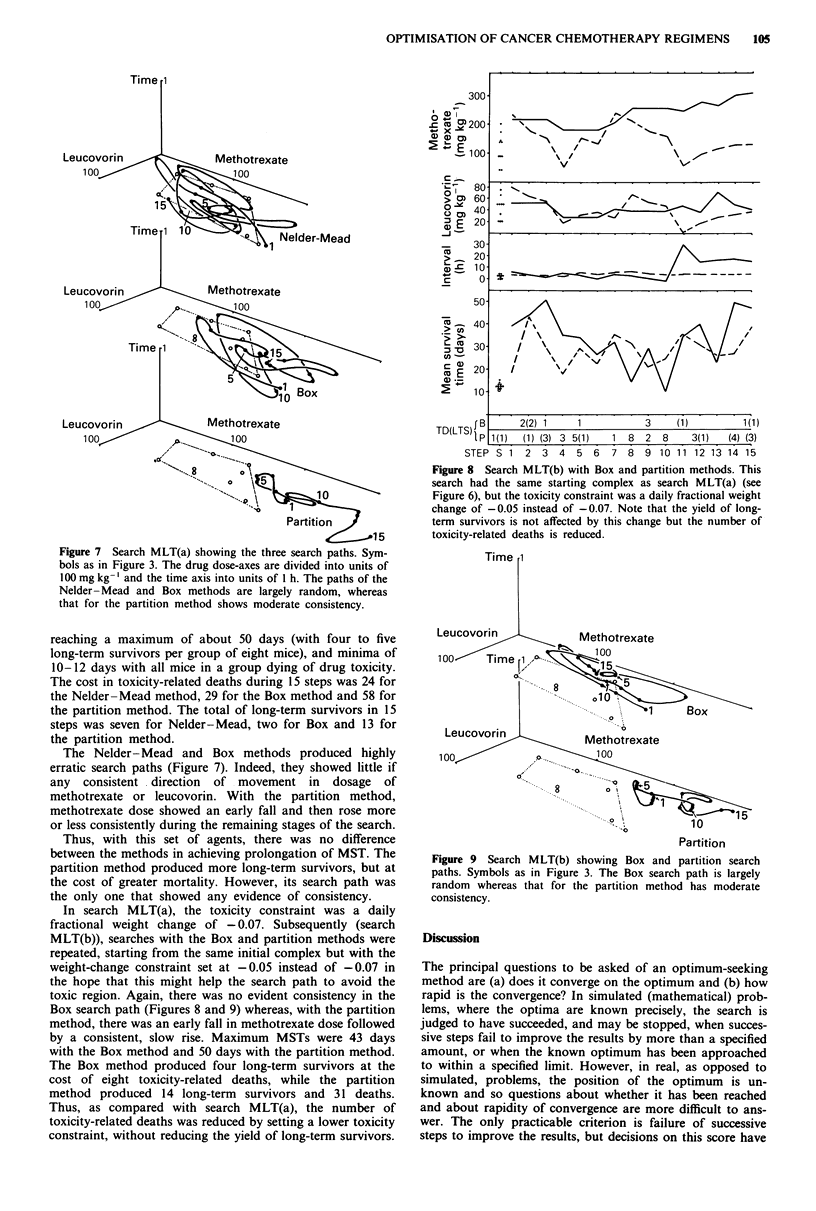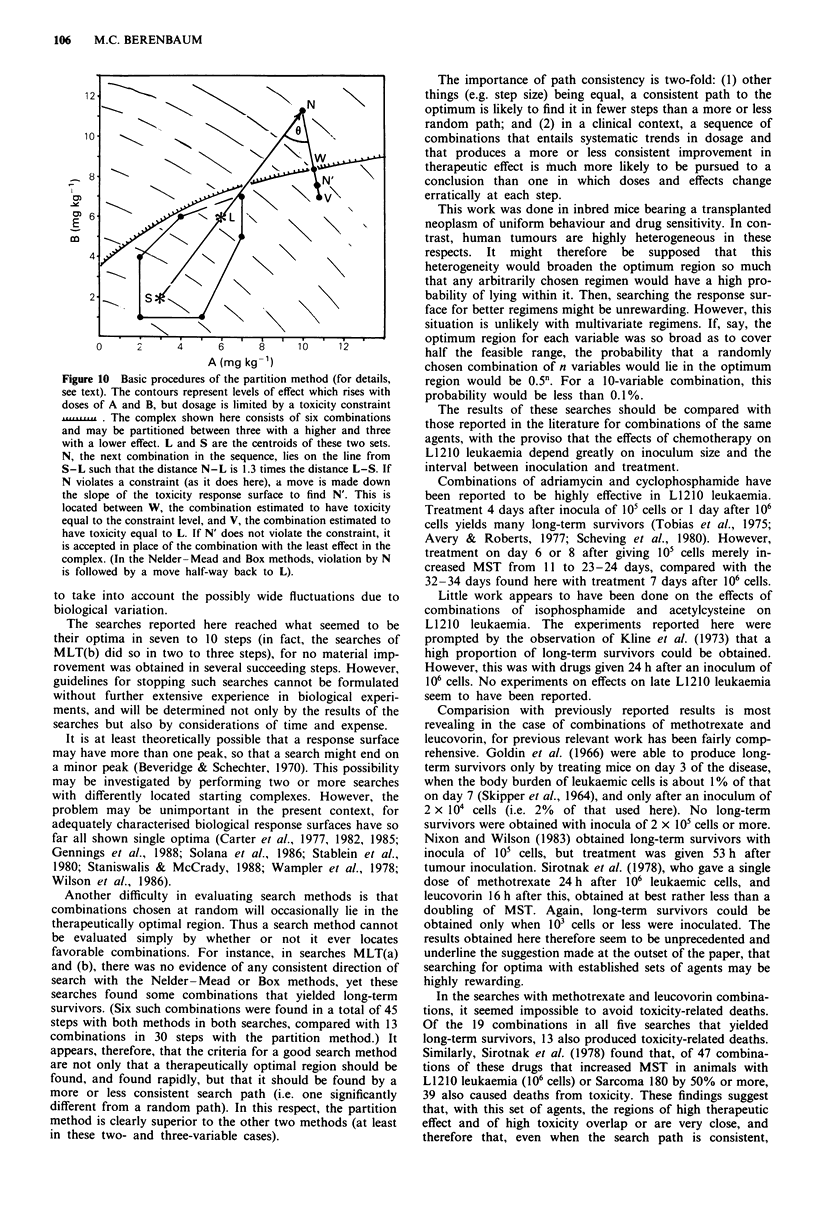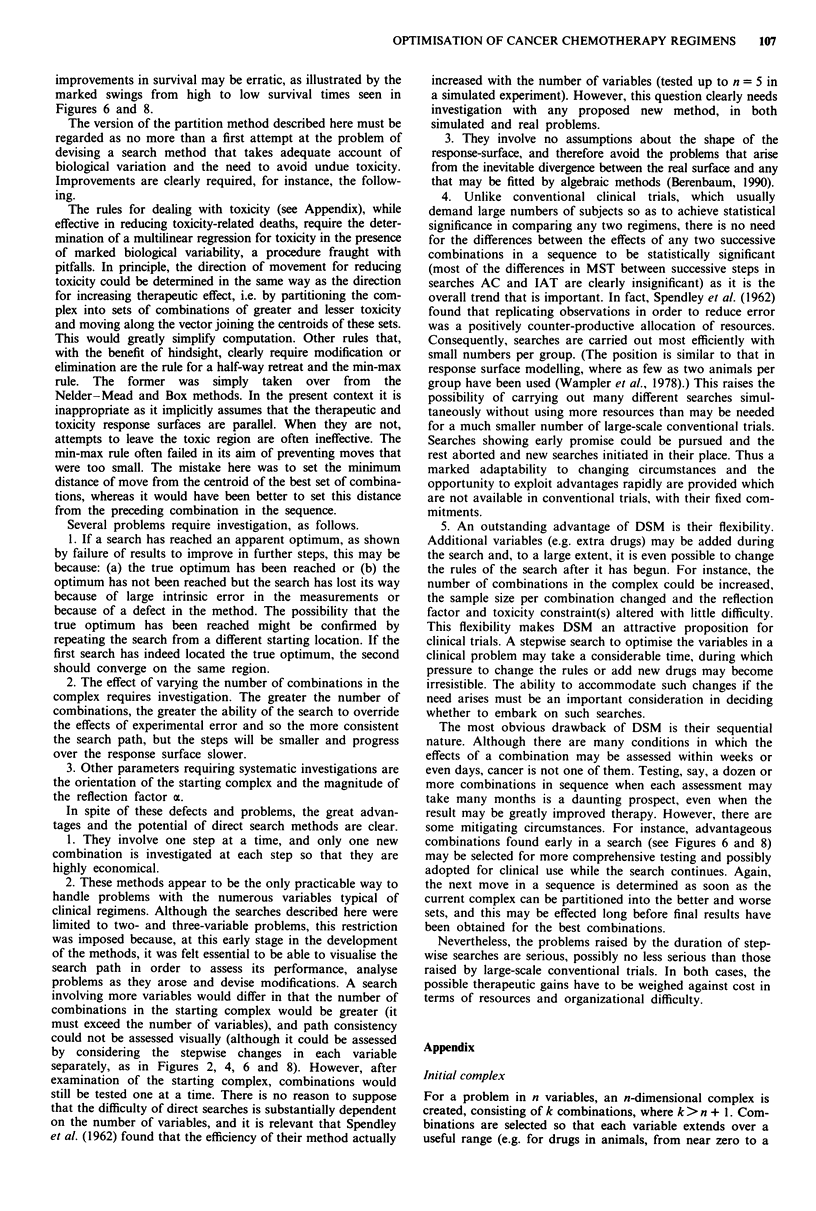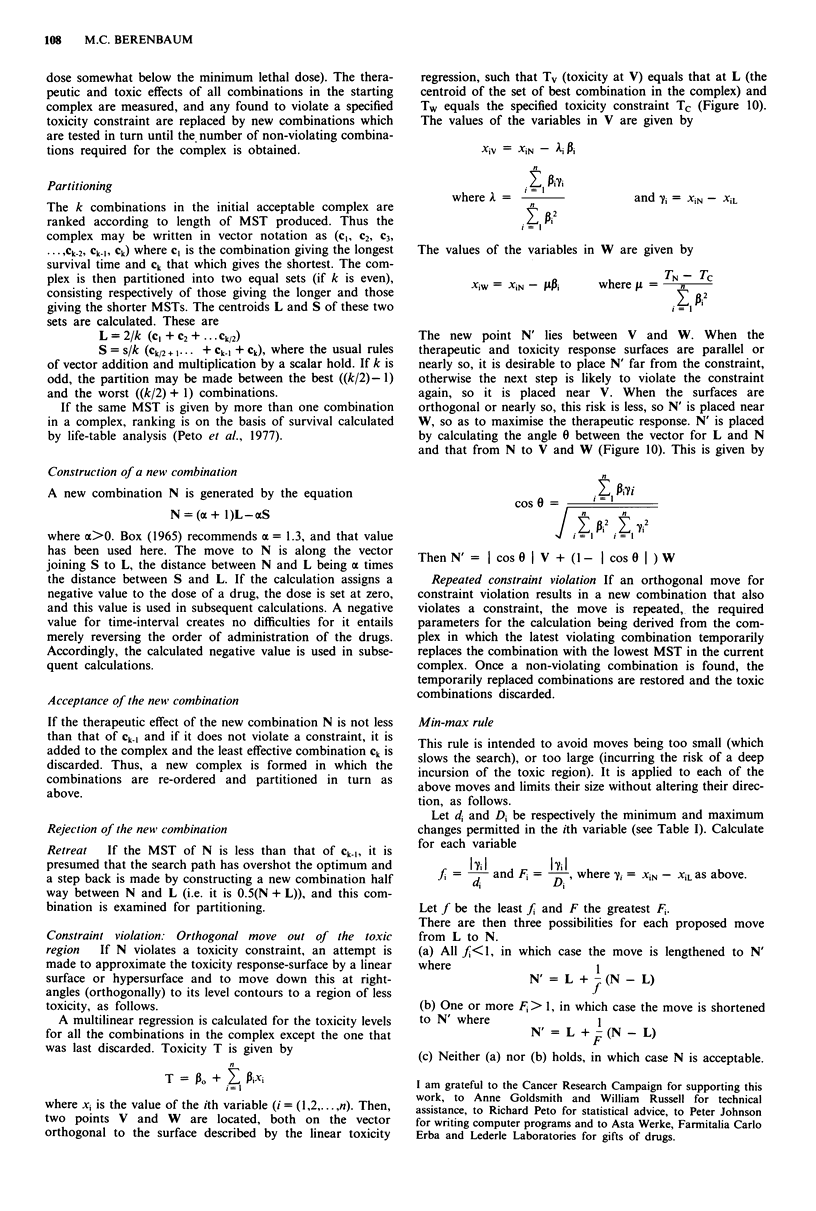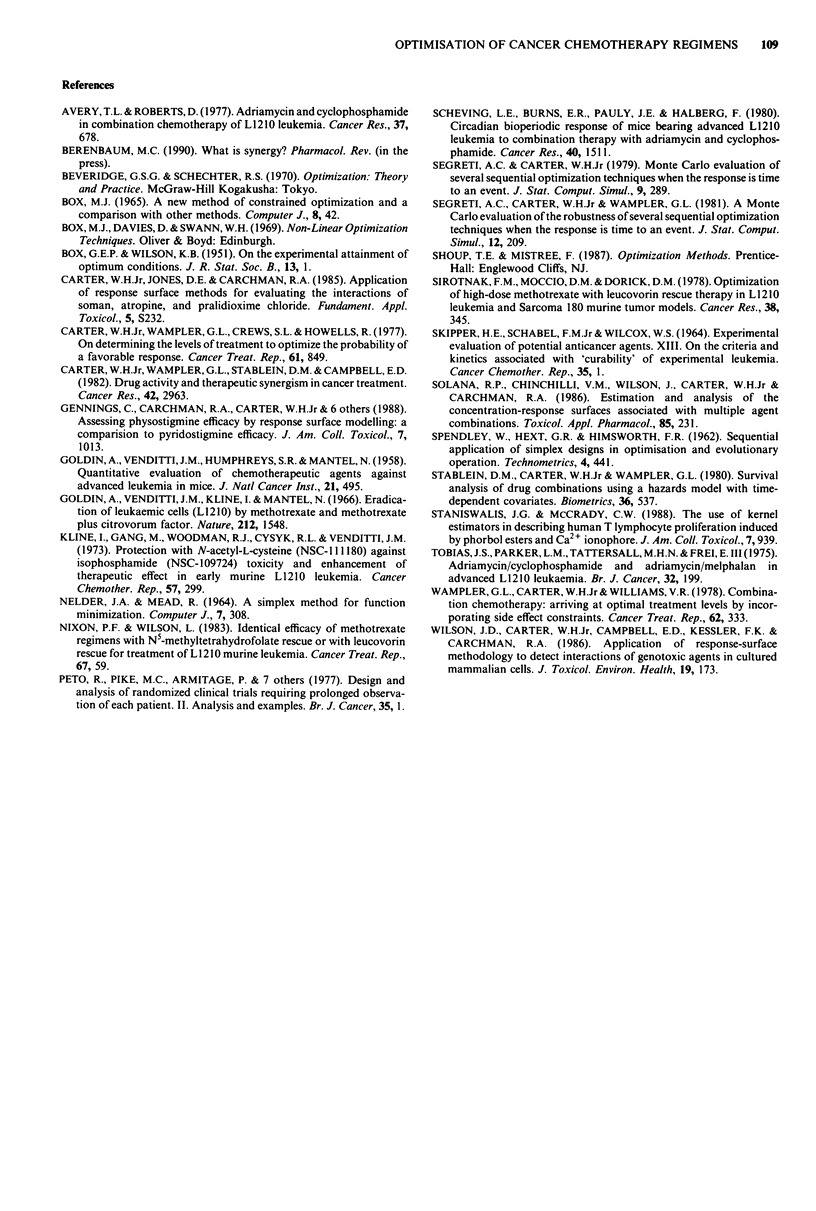# Direct search methods in the optimisation of cancer chemotherapy regimens.

**DOI:** 10.1038/bjc.1990.22

**Published:** 1990-01

**Authors:** M. C. Berenbaum

**Affiliations:** Department of Experimental Pathology, St Mary's Hospital Medical School, London, UK.

## Abstract

Current cancer chemotherapy regimens may involve 20-30 or more independent variables, each affecting therapeutic response and toxicity. With standard response surface modelling methods, finding the optimum combination with as few as 10 variables entails testing over 1,000 combinations, so these methods do not provide a feasible approach to such problems. However, they may be tackled by direct search methods (DSM), i.e. stepwise searches of the response surface. Experiments were carried out in advanced L1210 leukaemia treated with combinations of adriamycin with cyclophosphamide, isophosphamide with acetylcysteine and methotrexate with leucovorin. Two established DSM (Nelder-Mead and Box) were used, and a new method was designed to find consistent search paths in spite of wide biological variation. With methotrexate and leucovorin, DSM located combinations prolonging mean survival to 40-50 days (compared with 10.4 in controls) and giving high proportions of long-term survivors. These results were achieved with single injections of drugs given 7 days after injection of 10(6) leukaemic cells, i.e. 2-3 days before deaths began in untreated mice, and appear to be unprecedented with these agents. Searching for optimal combinations of established agents may be at least as rewarding as searching for new agents, and thus DSM may prove a powerful tool for improving the results of combination cancer chemotherapy.


*Br. J. Cancer* (1990), **61**, 101–109 © Macmillan Press Ltd., 1990

### Direct search methods in the optimisation of cancer chemotherapy regimens

M.C. Berenbaum

*Department of Experimental Pathology, St Mary's Hospital Medical School, London W2, UK.*

**Summary** Current cancer chemotherapy regimens may involve 20–30 or more independent variables, each affecting therapeutic response and toxicity. With standard response surface modelling methods, finding the optimum combination with as few as 10 variables entails testing over 1,000 combinations, so these methods do not provide a feasible approach to such problems. However, they may be tackled by direct search methods (DSM), i.e. stepwise searches of the response surface. Experiments were carried out in advanced L1210 leukaemia treated with combinations of adriamycin with cyclophosphamide, isophosphamide with acetylcysteine and methotrexate with leucovorin. Two established DSM (Nelder–Mead and Box) were used, and a new method was designed to find consistent search paths in spite of wide biological variation. With methotrexate and leucovorin, DSM located combinations prolonging mean survival to 40–50 days (compared with 10.4 in controls) and giving high proportions of long-term survivors. These results were achieved with single injections of drugs given 7 days after injection of $10^6$ leukaemic cells, i.e. 2–3 days before deaths began in untreated mice, and appear to be unprecedented with these agents. Searching for optimal combinations of established agents may be at least as rewarding as searching for new agents, and thus DSM may prove a powerful tool for improving the results of combination cancer chemotherapy.

Widely used cancer chemotherapy regimens may involve concurrent use of four to six or more agents and for each agent dose, number of doses and interval between doses may be independently varied. Furthermore, agents are commonly given in courses of defined length, and their length, number and the intervals between them may also be varied. Thus, up to six variables may be associated with each agent, so that a regimen of four to six agents may involve at least 20–30 variables, all of which may influence therapeutic and toxic effects.

The difficulty of finding the optimum combination of 20–30 variables becomes obvious when it is considered that when responses are not linear (and biological responses are usually not) each variable must be tested at at least three levels to determine its relation to response, and a full factorial design for a regimen of 20 variables would thus entail testing at least $3^{20}$ combinations. Investigators can hardly be blamed for avoiding such problems. However, the same considerations show that any complex drug regimen now in use is exceedingly unlikely to be optimal for that set of drugs; indeed it may be far from optimal, and there is therefore an unknown and possibly large gain to be made by searching for better combinations of the same set. This gain may in some cases be at least as great as that to be made by introducing a new agent.

There are two approaches to such problems, i.e. response surface modelling methods and direct search methods (DSM). In the former procedure, a predetermined number of combinations of predetermined composition is examined and the results fitted by an algebraic expression, usually a second-order polynomial, the maximum of which is found by standard mathematical methods. In DSM, after an initial set of combinations is tested, the response surface is searched one step (i.e. one combination) at a time, the location of each successive combination being determined by the results obtained with previous combinations. The main difficulty with response surface modelling is that the number of combinations that must be tested in order to fit a second-order function rises more or less exponentially with the number of variables. For example, the economical central composite design of Box and Wilson (1951) entails testing $2^n + 2n + 1$ combinations for a problem in $n$ variables. For a 10-variable problem, this means testing over 1,000 combinations. It is difficult to see how such procedures could be useful in the multivariate problems posed clinically.

A practical DSM procedure seems first to have been suggested by Spendley *et al.* (1962). Suppose we wish to find the best combination of agents A and B, varying doses only. In Figure 1 the axes represent drug doses and the contours represent the response surface (therapeutic effect). At the outset, no information is available about the shape of the response surface. First, we compare the effects of three arbitrarily chosen combinations (labelled 1, 2 and 3) which form the vertices of an equilateral triangle (i.e. a regular simplex in two dimensions). Combination 1 is found to have the least effect, so it is discarded and the triangle is reflected about the axis 2–3 to give combination 4 and a new triangle. Combina-

**Figure 1** Direct stepwise search of a response with combinations of drugs A and B (method of Spendley *et al.*, 1962). The contours represent levels of effect, and rise to a peak in the region of 7–8 mg kg$^{-1}$ A with 5–6 mg kg$^{-1}$ B. Initially, combinations 1, 2 and 3 are tested, which form the vertices of an equilateral triangle. Combination 1 has the least effect, so the triangle is reflected about axis 2–3 to give a new combination 4 and a new triangle. Combination 2 is the least effective here, and the next reflection gives combination 5. With successive reflections, the triangles climb the response-surface, turning with the contours, until the optimum is reached with combinations 14 and 15.

Received 5 April 1989; and in revised form 3 July 1989.

tion 2 is the least effective in this triangle so it is discarded in turn and the triangle reflected to give combination 5. Thus, successive moves climb the therapeutic response-surface until the region of the optimum is reached with combinations 14 and 15. To use this procedure for $n$ variables, a regular simplex with $n + 1$ vertices is used instead of a triangle (for example, if $n = 3$, the simplex is a tetrahedron).

Nelder and Mead (1964) modified this method to enable the simplex to expand or contract whenever its movement was favourable or unfavourable, and Box (1965) further improved it by increasing the number of vertices (creating what was called a 'complex') and by incorporating rules enabling the complex to retreat when it violated a predetermined limit or constraint. In the context of drug treatment, such constraints could include specified levels of toxicity. The Nelder–Mead method does not include an explicit rule for dealing with constraints, but it may be adapted for doing so by treating a violating combination as if it were the least effective combination in the simplex.

Direct search methods have been used successfully in industry and mathematics for many years, but the possibility that they might be useful in exploring combination cancer chemotherapy has so far been examined only in computer simulations (Segreti & Carter, 1979; Segreti *et al.*, 1981). The work reported here appears to be the first attempt to examine this possibility in practice.

Initially, the Nelder–Mead and Box methods were studied in computer simulations, using mathematically formulated problems with known solutions and artificially induced error, and it rapidly became clear that they coped poorly if at all with error of the magnitude typical of biological experiments. Both methods depend on identifying the least effective combination in the current set and, when error is high, there is a high probability that the wrong combination will be selected, and this acts in effect like a misleading signpost pointing in the wrong direction.

Furthermore, although both procedures incorporate rules enabling the search to escape from an unacceptably toxic region, neither is particularly efficient at this. The rules prescribe a partial reversal of the step that led into the toxic region. When the therapeutic and toxic response surfaces are not parallel (and they usually are not), many such reversals may be required before the non-toxic region is re-entered (Figure 10); in a clinical context, this repeated testing of toxic combinations would be a serious disadvantage.

Accordingly, these methods were modified in an attempt to overcome these drawbacks, and a new method, here called the partition method, was evolved as described below.

### Materials and methods

### *Mice*

$BDF_1$ (C57Bl × DBA/2) female mice weighing 17–21 g were obtained weekly from Olac Ltd, and were given food and water *ad libitum*.

### *Leukaemia*

The L1210 leukaemia, obtained from the Chester Beatty Research Institute, was passaged weekly as an ascites tumour by injection of $10^6$ cells intraperitoneally. For therapeutic tests, $10^6$ cells, suspended in phosphate-buffered saline, pH 7.4, were injected subcutaneously. This produced a small local tumour and a systemic disease that caused death in about 9–13 days (Goldin *et al.*, 1958, 1966).

### *Drugs*

Methotrexate sodium and leucovorin were obtained from Lederle Laboratories, cyclophosphamide and isophosphamide from Asta Werke, adriamycin from Farmitalia Carlo Erba and *N*-acetyl-L-cysteine from Sigma Laboratories. Drugs were dissolved in physiological saline within a few hours of use.

### *Regimens*

Cytotoxic drugs were given in single doses on day 7 after injection of leukaemic cells. Groups of eight mice were used for each combination and groups of 10 for untreated controls. Three drug regimens were used, as follows.

*Adriamycin-cyclophosphamide* Search AC, two-variable experiment. Single injections of each drug were given simultaneously (i.e. within 1 min of each other) into separate subcutaneous sites.

*Isophosphamide-acetylcysteine-time interval* Search IAT, three-variable experiments. A single injection of isophosphamide was given subcutaneously on day 7 and a single injection of acetylcysteine was given intraperitoneally at various times before or after this.

*Methotrexate-leucovorin-time interval* Searches MLT(a) and (b), three-variable experiment. A single injection of methotrexate was given subcutaneously on day 7 and a single injection of leucovorin subcutaneously at various times before or after this.

### *Therapeutic effect*

This was defined as mean survival time (MST). Mice surviving 60 days and showing no gross or microscopic evidence of leukaemia were classified as long-term survivors and, for the purpose of calculation, were assigned a survival time of 60 days. Consideration was given to evaluating therapeutic effect in terms of MSTs of treated animals expressed as fractions of concurrent control MSTs. However, the standard error of the mean MST in groups of control mice was only 1.5% of the mean (see below). With such small variation, it was decided not to use this correction as it would probably have reduced the accuracy of the results by allowing random errors in the controls to affect results for the treated groups (R. Peto, personal communication).

### *Toxic effects*

These were evaluated in two ways.

*Weight loss* Each group of mice was weighed daily from day 7 to day 11 inclusive (i.e. on days 0–4 after drug injection). The mean daily weight change over that period was expressed as a fraction of the weight on day 7.

*Death due to drug toxicity* There was usually little difficulty in deciding whether a mouse had died of toxicity or of leukaemia. Untreated mice usually died on about days 9–12 with livers grossly enlarged by leukaemic cell infiltration and weighing 11–13% or more (rarely less than 10%) of body weight, as compared with a normal liver weight of $4.53\% \pm 0.22\%$ (s.e.m.) ($n = 11$) of body weight. In histological sections, leukaemic cells usually occupied at least 75% of the section area, and mice could survive up to day 10–11 with 80–90% of the liver sectional area occupied by leukaemic cells. Accordingly, mice were judged to have died of toxicity rather than leukaemia if they died at or before 60 days with a liver weight less than 6% of body weight. Livers weighing 6–9% of body weight were examined histologically, and death was ascribed to toxicity if leukaemic cells occupied less than 50% of the area of the section.

### *Search methods*

The Nelder–Mead and Box methods are fully described in the original papers and in textbooks (Nelder & Mead, 1964; Box, 1965; Beveridge & Schechter, 1970; Box *et al.*, 1969; Shoup & Mistree, 1987). The partition method is described fully in the Appendix. Its principal differences from the other two methods were as follows. (1) The direction of steps up the therapeutic response surface was along the line joining the mean positions (centroids) of the most and the least effective sets of combinations in the complex, instead of along a line that depended on identifying the single least effective combination. This modification was intended to have the effect of smoothing out errors due to biological variability. (2) The direction of movement out of a toxic region was directly down the toxicity response surface instead of down the therapeutic surface.

*Search parameters*

The agents used in each search, the number $k$ of combinations in the complex, the maximum and minimum permitted moves and the toxicity constraints for these experiments are summarised in Table I.

### Results

The mean MST in 24 groups of 10 control mice inoculated over the period in which the initial complexes were being set up was 10.4 ± 0.17 (s.e.m.) days. Deaths began almost invariably on day 9 or 10 and all mice were usually dead by days 10–13.

*Adriamycin-cyclophosphamide*

Search AC is shown in Figures 2 and 3. The six arbitrarily chosen combinations in the starting complex increased MST from 10.4 to 12.6–17.4 days. All three search methods increased the MST to 24–26 days within the first four to six steps, and there was a further rise to 28–30 days (Nelder–Mead and Box methods) or to 32–34 days (partition method) in eight to 12 steps. There was no further consistent improvement after this. Only one long-term survivor was obtained in 15 steps in each of the Nelder–Mead and partition searches (at step 12 in each case).

With all three methods, several combinations were toxic. The total toxicity-related deaths in 15 steps (120 mice) were 40 for the Nelder–Mead method, 32 for Box and 19 for the partition method.

When the search paths were plotted graphically (Figure 3), all three searches tended to move away from the starting complex into a region of high doses of both drugs, but there was a clear difference between the rather erratic courses produced by the Nelder–Mead and Box methods and the more consistent course of the partition method.

Overall, the partition method caused fewer deaths from toxicity, had a more consistent search path and produced a slightly higher MST.

*Isophosphamide-acetylcysteine-time interval*

Search IAT is shown in Figures 4 and 5. The 10 combinations in the initial complex improved MST to 14–22 days. All three searches produced MSTs of 30–32 days (in nine steps with the Nelder–Mead, 11 with the Box and seven with the partition method), and subsequent moves did not improve on this. Deaths due to toxicity in 15 steps (120 mice)

**Table I** Search parameters, showing number $k$ of combinations in complex, toxicity constraint $T_c$ (fractional weight change), and maximum and minimum changes allowed in the variables.

| *Search* | *k* | $T_c$ | *Variables* | *Minimum change* | *Maximum change* |
|---|---|---|---|---|---|
| AC | 6 | −0.10 | Adriamycin (mg kg$^{-1}$) | 0.5 | 2.0 |
| | | | Cyclophosphamide (mg kg$^{-1}$) | 20.0 | 60.0 |
| IAT | 10 | −0.10 | Isophosphamide (mg kg$^{-1}$) | 25.0 | 100.0 |
| | | | Acetylcysteine (mg kg$^{-1}$) | 50.0 | 200.0 |
| | | | Time interval (min) | 15.0 | No limit |
| MLT(a) | 10 | −0.07 | Methotrexate (mg kg$^{-1}$) | 20.0 | 80.0 |
| | | | Leucovorin (mg kg$^{-1}$) | 10.0 | No limit |
| | | | Time interval (h) | 2.0 | No limit |
| MLT(b) | 10 | −0.05 | Methotrexate (mg kg$^{-1}$) | 20.0 | 80.0 |
| | | | Leucovorin (mg kg$^{-1}$) | 10.0 | No limit |
| | | | Time interval (h) | 2.0 | No limit |

**Figure 2** Search AC. Mice treated on day 7 with adriamycin and cyclophosphamide. Stepwise changes in the doses of the two drugs and in mean survival time (MST) are shown, and also the yield at each step of toxicity-related deaths (TD) and long-term survivors (LTS). Numbers of LTS are bracketed. Nelder–Mead method (NM) – – –, Box method (B) ......., partition method (P) ———. Starting complex, S. MST of controls O (standard error too small to be shown).

**Figure 3** Search AC showing search paths of Nelder–Mead, Box and partition methods. Combinations of starting complex O. Boundaries of starting complex ...... Combinations at steps 1, 5, 10 and 15 are indicated. Note the erratic courses of the Nelder–Mead and Box methods compared with the more consistent course of the partition method.

**Figure 4** Search IAT. Mice given isophosphamide on day 7. Stepwise changes in doses of isophosphamide and acetylcysteine and in time interval, in MST and in the yield of toxicity-related deaths and long-term survivors are shown. Symbols as in Figure 2.

**Figure 5** Search IAT showing the three search paths. Symbols as in Figure 3. The drug dose-axes are divided into units of $100\,mg\,kg^{-1}$ and the time axis into units of 100 min. Note the much more consistent search path of the partition method compared with the Nelder–Mead and Box methods. Symbols as in Figure 4.

were 11 for Nelder–Mead, 26 for Box and eight for the partition method.

The search path produced by the partition method was considerably more consistent than those produced by the Nelder–Mead or Box methods. This is clearly shown by the two dimensional representation in Figure 5 and by plotting the values of the three variables against step number as in Figure 4. The Nelder–Mead and Box methods showed some consistency in the progressive increase in isophosphamide dose but this consistency was considerably less than that shown by the smooth rise with the partition method.

Overall, there was little to choose between the three methods in terms of the maximum MST obtained, but the partition method achieved this maximum with less cost in toxicity and with a considerably more consistent search path.

A significant finding was that the partition method progressively reduced and virtually eliminated acetyclysteine from the regimen (Figure 4). This prompted an examination of the effects of isphosphamide on its own, which showed that this drug in doses of $520-620\,mg\,kg^{-1}$ gave MSTs of around 30 days, i.e. as good as that given by any combination of isophosphamide and acetylcysteine used here. It must be concluded that, in contrast to the results of Kline *et al.* (1973) discussed below, acetylcysteine does not improve on the results obtainable with isophosphamide alone when both drugs are given on day 7. Figure 4 shows that the steady reduction in dose of acetylcysteine (steps 1–9) was accompanied by a steady rise in MST, suggesting that this agent may have been positively deleterious in these experiments. Thus, the partition method not only optimises beneficial variables but actively eliminates those that are disadvantageous. The Box and Nelder–Mead methods did not do this in these experiments, probably because their search paths were too erratic.

### *Methotrexate-leucovorin-time interval*

Search MLT(a) is shown in Figures 6 and 7. The 10 combinations in the initial complex gave MSTs of 11.5–15.25 days. The MST fluctuated greatly during all three searches,

**Figure 6** Search MLT(a). Mice given methotrexate on day 7. Stepwise changes in doses of methotrexate and leucovorin and time interval, and in the yield of toxicity-related deaths and long-term survivors are shown. Symbols as in Figure 2. Note the wide swings in MST due largely to differences in the number of toxicity-related deaths. Combinations yielding long-term survivors are usually also toxic. The stepwise changes in methotrexate and leucovorin dosage with the Nelder–Mead and Box methods appear to be largely random.

**Figure 7** Search MLT(a) showing the three search paths. Symbols as in Figure 3. The drug dose-axes are divided into units of 100 mg $kg^{-1}$ and the time axis into units of 1 h. The paths of the Nelder–Mead and Box methods are largely random, whereas that for the partition method shows moderate consistency.

**Figure 8** Search MLT(b) with Box and partition methods. This search had the same starting complex as search MLT(a) (see Figure 6), but the toxicity constraint was a daily fractional weight change of −0.05 instead of −0.07. Note that the yield of long-term survivors is not affected by this change but the number of toxicity-related deaths is reduced.

**Figure 9** Search MLT(b) showing Box and partition search paths. Symbols as in Figure 3. The Box search path is largely random whereas that for the partition method has moderate consistency.

reaching a maximum of about 50 days (with four to five long-term survivors per group of eight mice), and minima of 10–12 days with all mice in a group dying of drug toxicity. The cost in toxicity-related deaths during 15 steps was 24 for the Nelder–Mead method, 29 for the Box method and 58 for the partition method. The total of long-term survivors in 15 steps was seven for Nelder–Mead, two for Box and 13 for the partition method.

The Nelder–Mead and Box methods produced highly erratic search paths (Figure 7). Indeed, they showed little if any consistent direction of movement in dosage of methotrexate or leucovorin. With the partition method, methotrexate dose showed an early fall and then rose more or less consistently during the remaining stages of the search.

Thus, with this set of agents, there was no difference between the methods in achieving prolongation of MST. The partition method produced more long-term survivors, but at the cost of greater mortality. However, its search path was the only one that showed any evidence of consistency.

In search MLT(a), the toxicity constraint was a daily fractional weight change of −0.07. Subsequently (search MLT(b)), searches with the Box and partition methods were repeated, starting from the same initial complex but with the weight-change constraint set at −0.05 instead of −0.07 in the hope that this might help the search path to avoid the toxic region. Again, there was no evident consistency in the Box search path (Figures 8 and 9) whereas, with the partition method, there was an early fall in methotrexate dose followed by a consistent, slow rise. Maximum MSTs were 43 days with the Box method and 50 days with the partition method. The Box method produced four long-term survivors at the cost of eight toxicity-related deaths, while the partition method produced 14 long-term survivors and 31 deaths. Thus, as compared with search MLT(a), the number of toxicity-related deaths was reduced by setting a lower toxicity constraint, without reducing the yield of long-term survivors.

### Discussion

The principal questions to be asked of an optimum-seeking method are (a) does it converge on the optimum and (b) how rapid is the convergence? In simulated (mathematical) problems, where the optima are known precisely, the search is judged to have succeeded, and may be stopped, when successive steps fail to improve the results by more than a specified amount, or when the known optimum has been approached to within a specified limit. However, in real, as opposed to simulated, problems, the position of the optimum is unknown and so questions about whether it has been reached and about rapidity of convergence are more difficult to answer. The only practicable criterion is failure of successive steps to improve the results, but decisions on this score have

**Figure 10** Basic procedures of the partition method (for details, see text). The contours represent levels of effect which rises with doses of A and B, but dosage is limited by a toxicity constraint . The complex shown here consists of six combinations and may be partitioned between three with a higher and three with a lower effect. **L** and **S** are the centroids of these two sets. **N**, the next combination in the sequence, lies on the line from **S–L** such that the distance **N–L** is 1.3 times the distance **L–S**. If **N** violates a constraint (as it does here), a move is made down the slope of the toxicity response surface to find **N'**. This is located between **W**, the combination estimated to have toxicity equal to the constraint level, and **V**, the combination estimated to have toxicity equal to **L**. If **N'** does not violate the constraint, it is accepted in place of the combination with the least effect in the complex. (In the Nelder–Mead and Box methods, violation by **N** is followed by a move half-way back to **L**).

to take into account the possibly wide fluctuations due to biological variation.

The searches reported here reached what seemed to be their optima in seven to 10 steps (in fact, the searches of MLT(b) did so in two to three steps), for no material improvement was obtained in several succeeding steps. However, guidelines for stopping such searches cannot be formulated without further extensive experience in biological experiments, and will be determined not only by the results of the searches but also by considerations of time and expense.

It is at least theoretically possible that a response surface may have more than one peak, so that a search might end on a minor peak (Beveridge & Schechter, 1970). This possibility may be investigated by performing two or more searches with differently located starting complexes. However, the problem may be unimportant in the present context, for adequately characterised biological response surfaces have so far all shown single optima (Carter *et al.*, 1977, 1982, 1985; Gennings *et al.*, 1988; Solana *et al.*, 1986; Stablein *et al.*, 1980; Staniswalis & McCrady, 1988; Wampler *et al.*, 1978; Wilson *et al.*, 1986).

Another difficulty in evaluating search methods is that combinations chosen at random will occasionally lie in the therapeutically optimal region. Thus a search method cannot be evaluated simply by whether or not it ever locates favorable combinations. For instance, in searches MLT(a) and (b), there was no evidence of any consistent direction of search with the Nelder–Mead or Box methods, yet these searches found some combinations that yielded long-term survivors. (Six such combinations were found in a total of 45 steps with both methods in both searches, compared with 13 combinations in 30 steps with the partition method.) It appears, therefore, that the criteria for a good search method are not only that a therapeutically optimal region should be found, and found rapidly, but that it should be found by a more or less consistent search path (i.e. one significantly different from a random path). In this respect, the partition method is clearly superior to the other two methods (at least in these two- and three-variable cases).

The importance of path consistency is two-fold: (1) other things (e.g. step size) being equal, a consistent path to the optimum is likely to find it in fewer steps than a more or less random path; and (2) in a clinical context, a sequence of combinations that entails systematic trends in dosage and that produces a more or less consistent improvement in therapeutic effect is much more likely to be pursued to a conclusion than one in which doses and effects change erratically at each step.

This work was done in inbred mice bearing a transplanted neoplasm of uniform behaviour and drug sensitivity. In contrast, human tumours are highly heterogeneous in these respects. It might therefore be supposed that this heterogeneity would broaden the optimum region so much that any arbitrarily chosen regimen would have a high probability of lying within it. Then, searching the response surface for better regimens might be unrewarding. However, this situation is unlikely with multivariate regimens. If, say, the optimum region for each variable was so broad as to cover half the feasible range, the probability that a randomly chosen combination of $n$ variables would lie in the optimum region would be $0.5^n$. For a 10-variable combination, this probability would be less than 0.1%.

The results of these searches should be compared with those reported in the literature for combinations of the same agents, with the proviso that the effects of chemotherapy on L1210 leukaemia depend greatly on inoculum size and the interval between inoculation and treatment.

Combinations of adriamycin and cyclophosphamide have been reported to be highly effective in L1210 leukaemia. Treatment 4 days after inocula of $10^5$ cells or 1 day after $10^6$ cells yields many long-term survivors (Tobias *et al.*, 1975; Avery & Roberts, 1977; Scheving *et al.*, 1980). However, treatment on day 6 or 8 after giving $10^5$ cells merely increased MST from 11 to 23–24 days, compared with the 32–34 days found here with treatment 7 days after $10^6$ cells.

Little work appears to have been done on the effects of combinations of isophosphamide and acetylcysteine on L1210 leukaemia. The experiments reported here were prompted by the observation of Kline *et al.* (1973) that a high proportion of long-term survivors could be obtained. However, this was with drugs given 24 h after an inoculum of $10^6$ cells. No experiments on effects on late L1210 leukaemia seem to have been reported.

Comparision with previously reported results is most revealing in the case of combinations of methotrexate and leucovorin, for previous relevant work has been fairly comprehensive. Goldin *et al.* (1966) were able to produce long-term survivors only by treating mice on day 3 of the disease, when the body burden of leukaemic cells is about 1% of that on day 7 (Skipper *et al.*, 1964), and only after an inoculum of $2 \times 10^4$ cells (i.e. 2% of that used here). No long-term survivors were obtained with inocula of $2 \times 10^5$ cells or more. Nixon and Wilson (1983) obtained long-term survivors with inocula of $10^5$ cells, but treatment was given 53 h after tumour inoculation. Sirotnak *et al.* (1978), who gave a single dose of methotrexate 24 h after $10^6$ leukaemic cells, and leucovorin 16 h after this, obtained at best rather less than a doubling of MST. Again, long-term survivors could be obtained only when $10^3$ cells or less were inoculated. The results obtained here therefore seem to be unprecedented and underline the suggestion made at the outset of the paper, that searching for optima with established sets of agents may be highly rewarding.

In the searches with methotrexate and leucovorin combinations, it seemed impossible to avoid toxicity-related deaths. Of the 19 combinations in all five searches that yielded long-term survivors, 13 also produced toxicity-related deaths. Similarly, Sirotnak *et al.* (1978) found that, of 47 combinations of these drugs that increased MST in animals with L1210 leukaemia ($10^6$ cells) or Sarcoma 180 by 50% or more, 39 also caused deaths from toxicity. These findings suggest that, with this set of agents, the regions of high therapeutic effect and of high toxicity overlap or are very close, and therefore that, even when the search path is consistent, improvements in survival may be erratic, as illustrated by the marked swings from high to low survival times seen in Figures 6 and 8.

The version of the partition method described here must be regarded as no more than a first attempt at the problem of devising a search method that takes adequate account of biological variation and the need to avoid undue toxicity. Improvements are clearly required, for instance, the following.

The rules for dealing with toxicity (see Appendix), while effective in reducing toxicity-related deaths, require the determination of a multilinear regression for toxicity in the presence of marked biological variability, a procedure fraught with pitfalls. In principle, the direction of movement for reducing toxicity could be determined in the same way as the direction for increasing therapeutic effect, i.e. by partitioning the complex into sets of combinations of greater and lesser toxicity and moving along the vector joining the centroids of these sets. This would greatly simplify computation. Other rules that, with the benefit of hindsight, clearly require modification or elimination are the rule for a half-way retreat and the min-max rule. The former was simply taken over from the Nelder–Mead and Box methods. In the present context it is inappropriate as it implicitly assumes that the therapeutic and toxicity response surfaces are parallel. When they are not, attempts to leave the toxic region are often ineffective. The min-max rule often failed in its aim of preventing moves that were too small. The mistake here was to set the minimum distance of move from the centroid of the best set of combinations, whereas it would have been better to set this distance from the preceding combination in the sequence.

Several problems require investigation, as follows.

1. If a search has reached an apparent optimum, as shown by failure of results to improve in further steps, this may be because: (a) the true optimum has been reached or (b) the optimum has not been reached but the search has lost its way because of large intrinsic error in the measurements or because of a defect in the method. The possibility that the true optimum has been reached might be confirmed by repeating the search from a different starting location. If the first search has indeed located the true optimum, the second should converge on the same region.

2. The effect of varying the number of combinations in the complex requires investigation. The greater the number of combinations, the greater the ability of the search to override the effects of experimental error and so the more consistent the search path, but the steps will be smaller and progress over the response surface slower.

3. Other parameters requiring systematic investigations are the orientation of the starting complex and the magnitude of the reflection factor $\alpha$.

In spite of these defects and problems, the great advantages and the potential of direct search methods are clear.

1. They involve one step at a time, and only one new combination is investigated at each step so that they are highly economical.

2. These methods appear to be the only practicable way to handle problems with the numerous variables typical of clinical regimens. Although the searches described here were limited to two- and three-variable problems, this restriction was imposed because, at this early stage in the development of the methods, it was felt essential to be able to visualise the search path in order to assess its performance, analyse problems as they arose and devise modifications. A search involving more variables would differ in that the number of combinations in the starting complex would be greater (it must exceed the number of variables), and path consistency could not be assessed visually (although it could be assessed by considering the stepwise changes in each variable separately, as in Figures 2, 4, 6 and 8). However, after examination of the starting complex, combinations would still be tested one at a time. There is no reason to suppose that the difficulty of direct searches is substantially dependent on the number of variables, and it is relevant that Spendley *et al.* (1962) found that the efficiency of their method actually increased with the number of variables (tested up to $n = 5$ in a simulated experiment). However, this question clearly needs investigation with any proposed new method, in both simulated and real problems.

3. They involve no assumptions about the shape of the response-surface, and therefore avoid the problems that arise from the inevitable divergence between the real surface and any that may be fitted by algebraic methods (Berenbaum, 1990).

4. Unlike conventional clinical trials, which usually demand large numbers of subjects so as to achieve statistical significance in comparing any two regimens, there is no need for the differences between the effects of any two successive combinations in a sequence to be statistically significant (most of the differences in MST between successive steps in searches AC and IAT are clearly insignificant) as it is the overall trend that is important. In fact, Spendley *et al.* (1962) found that replicating observations in order to reduce error was a positively counter-productive allocation of resources. Consequently, searches are carried out most efficiently with small numbers per group. (The position is similar to that in response surface modelling, where as few as two animals per group have been used (Wampler *et al.*, 1978).) This raises the possibility of carrying out many different searches simultaneously without using more resources than may be needed for a much smaller number of large-scale conventional trials. Searches showing early promise could be pursued and the rest aborted and new searches initiated in their place. Thus a marked adaptability to changing circumstances and the opportunity to exploit advantages rapidly are provided which are not available in conventional trials, with their fixed commitments.

5. An outstanding advantage of DSM is their flexibility. Additional variables (e.g. extra drugs) may be added during the search and, to a large extent, it is even possible to change the rules of the search after it has begun. For instance, the number of combinations in the complex could be increased, the sample size per combination changed and the reflection factor and toxicity constraint(s) altered with little difficulty. This flexibility makes DSM an attractive proposition for clinical trials. A stepwise search to optimise the variables in a clinical problem may take a considerable time, during which pressure to change the rules or add new drugs may become irresistible. The ability to accommodate such changes if the need arises must be an important consideration in deciding whether to embark on such searches.

The most obvious drawback of DSM is their sequential nature. Although there are many conditions in which the effects of a combination may be assessed within weeks or even days, cancer is not one of them. Testing, say, a dozen or more combinations in sequence when each assessment may take many months is a daunting prospect, even when the result may be greatly improved therapy. However, there are some mitigating circumstances. For instance, advantageous combinations found early in a search (see Figures 6 and 8) may be selected for more comprehensive testing and possibly adopted for clinical use while the search continues. Again, the next move in a sequence is determined as soon as the current complex can be partitioned into the better and worse sets, and this may be effected long before final results have been obtained for the best combinations.

Nevertheless, the problems raised by the duration of stepwise searches are serious, possibly no less serious than those raised by large-scale conventional trials. In both cases, the possible therapeutic gains have to be weighed against cost in terms of resources and organizational difficulty.

### Appendix

### *Initial complex*

For a problem in $n$ variables, an $n$-dimensional complex is created, consisting of $k$ combinations, where $k > n + 1$. Combinations are selected so that each variable extends over a useful range (e.g. for drugs in animals, from near zero to a dose somewhat below the minimum lethal dose). The therapeutic and toxic effects of all combinations in the starting complex are measured, and any found to violate a specified toxicity constraint are replaced by new combinations which are tested in turn until the number of non-violating combinations required for the complex is obtained.

*Partitioning*

The $k$ combinations in the initial acceptable complex are ranked according to length of MST produced. Thus the complex may be written in vector notation as ($\mathbf{c}_1$, $\mathbf{c}_2$, $\mathbf{c}_3$, ...,$\mathbf{c}_{k-2}$, $\mathbf{c}_{k-1}$, $\mathbf{c}_k$) where $\mathbf{c}_1$ is the combination giving the longest survival time and $\mathbf{c}_k$ that which gives the shortest. The complex is then partitioned into two equal sets (if $k$ is even), consisting respectively of those giving the longer and those giving the shorter MSTs. The centroids **L** and **S** of these two sets are calculated. These are

$\mathbf{L} = 2/k\ (\mathbf{c}_1 + \mathbf{c}_2 + \ldots \mathbf{c}_{k/2})$

$\mathbf{S} = s/k\ (\mathbf{c}_{k/2+1} \ldots + \mathbf{c}_{k-1} + \mathbf{c}_k)$, where the usual rules of vector addition and multiplication by a scalar hold. If $k$ is odd, the partition may be made between the best $((k/2)-1)$ and the worst $((k/2)+1)$ combinations.

If the same MST is given by more than one combination in a complex, ranking is on the basis of survival calculated by life-table analysis (Peto *et al.*, 1977).

*Construction of a new combination*

A new combination **N** is generated by the equation

$$\mathbf{N} = (\alpha + 1)\mathbf{L} - \alpha\mathbf{S}$$

where $\alpha > 0$. Box (1965) recommends $\alpha = 1.3$, and that value has been used here. The move to **N** is along the vector joining **S** to **L**, the distance between **N** and **L** being $\alpha$ times the distance between **S** and **L**. If the calculation assigns a negative value to the dose of a drug, the dose is set at zero, and this value is used in subsequent calculations. A negative value for time-interval creates no difficulties for it entails merely reversing the order of administration of the drugs. Accordingly, the calculated negative value is used in subsequent calculations.

*Acceptance of the new combination*

If the therapeutic effect of the new combination **N** is not less than that of $\mathbf{c}_{k-1}$ and if it does not violate a constraint, it is added to the complex and the least effective combination $\mathbf{c}_k$ is discarded. Thus, a new complex is formed in which the combinations are re-ordered and partitioned in turn as above.

*Rejection of the new combination*

*Retreat* If the MST of **N** is less than that of $\mathbf{c}_{k-1}$, it is presumed that the search path has overshot the optimum and a step back is made by constructing a new combination half way between **N** and **L** (i.e. it is $0.5(\mathbf{N} + \mathbf{L})$), and this combination is examined for partitioning.

*Constraint violation: Orthogonal move out of the toxic region* If **N** violates a toxicity constraint, an attempt is made to approximate the toxicity response-surface by a linear surface or hypersurface and to move down this at right-angles (orthogonally) to its level contours to a region of less toxicity, as follows.

A multilinear regression is calculated for the toxicity levels for all the combinations in the complex except the one that was last discarded. Toxicity T is given by

$$T = \beta_o + \sum_{i=1}^{n} \beta_i x_i$$

where $x_i$ is the value of the $i$th variable ($i = (1,2,\ldots,n)$. Then, two points **V** and **W** are located, both on the vector orthogonal to the surface described by the linear toxicity regression, such that $T_V$ (toxicity at **V**) equals that at **L** (the centroid of the set of best combination in the complex) and $T_W$ equals the specified toxicity constraint $T_C$ (Figure 10). The values of the variables in **V** are given by

$$x_{iV} = x_{iN} - \lambda_i \beta_i$$

$$\text{where } \lambda = \frac{\sum_{i=1}^{n} \beta_i \gamma_i}{\sum_{i=1}^{n} \beta_i^2} \qquad \text{and } \gamma_i = x_{iN} - x_{iL}$$

The values of the variables in **W** are given by

$$x_{iW} = x_{iN} - \mu\beta_i \qquad \text{where } \mu = \frac{T_N - T_C}{\sum_{i=1}^{n} \beta_i^2}$$

The new point **N'** lies between **V** and **W**. When the therapeutic and toxicity response surfaces are parallel or nearly so, it is desirable to place **N'** far from the constraint, otherwise the next step is likely to violate the constraint again, so it is placed near **V**. When the surfaces are orthogonal or nearly so, this risk is less, so **N'** is placed near **W**, so as to maximise the therapeutic response. **N'** is placed by calculating the angle $\theta$ between the vector for **L** and **N** and that from **N** to **V** and **W** (Figure 10). This is given by

$$\cos\theta = \frac{\sum_{i=1}^{n} \beta_i \gamma_i}{\sqrt{\sum_{i=1}^{n} \beta_i^2 \sum_{i=1}^{n} \gamma_i^2}}$$

Then $\mathbf{N}' = |\cos\theta|\,\mathbf{V} + (1 - |\cos\theta|)\,\mathbf{W}$

*Repeated constraint violation* If an orthogonal move for constraint violation results in a new combination that also violates a constraint, the move is repeated, the required parameters for the calculation being derived from the complex in which the latest violating combination temporarily replaces the combination with the lowest MST in the current complex. Once a non-violating combination is found, the temporarily replaced combinations are restored and the toxic combinations discarded.

*Min-max rule*

This rule is intended to avoid moves being too small (which slows the search), or too large (incurring the risk of a deep incursion of the toxic region). It is applied to each of the above moves and limits their size without altering their direction, as follows.

Let $d_i$ and $D_i$ be respectively the minimum and maximum changes permitted in the $i$th variable (see Table I). Calculate for each variable

$$f_i = \frac{|\gamma_i|}{d_i} \text{ and } F_i = \frac{|\gamma_i|}{D_i}, \text{ where } \gamma_i = x_{iN} - x_{iL} \text{ as above.}$$

Let $f$ be the least $f_i$ and $F$ the greatest $F_i$.

There are then three possibilities for each proposed move from **L** to **N**.

(a) All $f_i < 1$, in which case the move is lengthened to **N'** where

$$\mathbf{N}' = \mathbf{L} + \frac{1}{f}(\mathbf{N} - \mathbf{L})$$

(b) One or more $F_i > 1$, in which case the move is shortened to **N'** where

$$\mathbf{N}' = \mathbf{L} + \frac{1}{F}(\mathbf{N} - \mathbf{L})$$

(c) Neither (a) nor (b) holds, in which case **N** is acceptable.

I am grateful to the Cancer Research Campaign for supporting this work, to Anne Goldsmith and William Russell for technical assistance, to Richard Peto for statistical advice, to Peter Johnson for writing computer programs and to Asta Werke, Farmitalia Carlo Erba and Lederle Laboratories for gifts of drugs.

### References

AVERY, T.L. & ROBERTS, D. (1977). Adriamycin and cyclophosphamide in combination chemotherapy of L1210 leukemia. *Cancer Res.*, **37**, 678.

BERENBAUM, M.C. (1990). What is synergy? *Pharmacol. Rev.* (in the press).

BEVERIDGE, G.S.G. & SCHECHTER, R.S. (1970). *Optimization: Theory and Practice*. McGraw-Hill Kogakusha: Tokyo.

BOX, M.J. (1965). A new method of constrained optimization and a comparison with other methods. *Computer J.*, **8**, 42.

BOX, M.J., DAVIES, D. & SWANN, W.H. (1969). *Non-Linear Optimization Techniques*. Oliver & Boyd: Edinburgh.

BOX, G.E.P. & WILSON, K.B. (1951). On the experimental attainment of optimum conditions. *J. R. Stat. Soc. B.*, **13**, 1.

CARTER, W.H.Jr, JONES, D.E. & CARCHMAN, R.A. (1985). Application of response surface methods for evaluating the interactions of soman, atropine, and pralidioxime chloride. *Fundament. Appl. Toxicol.*, **5**, S232.

CARTER, W.H.Jr, WAMPLER, G.L., CREWS, S.L. & HOWELLS, R. (1977). On determining the levels of treatment to optimize the probability of a favorable response. *Cancer Treat. Rep.*, **61**, 849.

CARTER, W.H.Jr, WAMPLER, G.L., STABLEIN, D.M. & CAMPBELL, E.D. (1982). Drug activity and therapeutic synergism in cancer treatment. *Cancer Res.*, **42**, 2963.

GENNINGS, C., CARCHMAN, R.A., CARTER, W.H.Jr & 6 others (1988). Assessing physostigmine efficacy by response surface modelling: a comparision to pyridostigmine efficacy. *J. Am. Coll. Toxicol.*, **7**, 1013.

GOLDIN, A., VENDITTI, J.M., HUMPHREYS, S.R. & MANTEL, N. (1958). Quantitative evaluation of chemotherapeutic agents against advanced leukemia in mice. *J. Natl Cancer Inst.*, **21**, 495.

GOLDIN, A., VENDITTI, J.M., KLINE, I. & MANTEL, N. (1966). Eradication of leukaemic cells (L1210) by methotrexate and methotrexate plus citrovorum factor. *Nature*, **212**, 1548.

KLINE, I., GANG, M., WOODMAN, R.J., CYSYK, R.L. & VENDITTI, J.M. (1973). Protection with *N*-acetyl-L-cysteine (NSC-111180) against isophosphamide (NSC-109724) toxicity and enhancement of therapeutic effect in early murine L1210 leukemia. *Cancer Chemother. Rep.*, **57**, 299.

NELDER, J.A. & MEAD, R. (1964). A simplex method for function minimization. *Computer J.*, **7**, 308.

NIXON, P.F. & WILSON, L. (1983). Identical efficacy of methotrexate regimens with $N^5$-methyltetrahydrofolate rescue or with leucovorin rescue for treatment of L1210 murine leukemia. *Cancer Treat. Rep.*, **67**, 59.

PETO, R., PIKE, M.C., ARMITAGE, P. & 7 others (1977). Design and analysis of randomized clinical trials requiring prolonged observation of each patient. II. Analysis and examples. *Br. J. Cancer*, **35**, 1.

SCHEVING, L.E., BURNS, E.R., PAULY, J.E. & HALBERG, F. (1980). Circadian bioperiodic response of mice bearing advanced L1210 leukemia to combination therapy with adriamycin and cyclophosphamide. *Cancer Res.*, **40**, 1511.

SEGRETI, A.C. & CARTER, W.H.Jr (1979). Monte Carlo evaluation of several sequential optimization techniques when the response is time to an event. *J. Stat. Comput. Simul.*, **9**, 289.

SEGRETI, A.C., CARTER, W.H.Jr & WAMPLER, G.L. (1981). A Monte Carlo evaluation of the robustness of several sequential optimization techniques when the response is time to an event. *J. Stat. Comput. Simul.*, **12**, 209.

SHOUP, T.E. & MISTREE, F. (1987). *Optimization Methods*. Prentice-Hall: Englewood Cliffs, NJ.

SIROTNAK, F.M., MOCCIO, D.M. & DORICK, D.M. (1978). Optimization of high-dose methotrexate with leucovorin rescue therapy in L1210 leukemia and Sarcoma 180 murine tumor models. *Cancer Res.*, **38**, 345.

SKIPPER, H.E., SCHABEL, F.M.Jr & WILCOX, W.S. (1964). Experimental evaluation of potential anticancer agents. XIII. On the criteria and kinetics associated with 'curability' of experimental leukemia. *Cancer Chemother. Rep.*, **35**, 1.

SOLANA, R.P., CHINCHILLI, V.M., WILSON, J., CARTER, W.H.Jr & CARCHMAN, R.A. (1986). Estimation and analysis of the concentration-response surfaces associated with multiple agent combinations. *Toxicol. Appl. Pharmacol.*, **85**, 231.

SPENDLEY, W., HEXT, G.R. & HIMSWORTH, F.R. (1962). Sequential application of simplex designs in optimisation and evolutionary operation. *Technometrics*, **4**, 441.

STABLEIN, D.M., CARTER, W.H.Jr & WAMPLER, G.L. (1980). Survival analysis of drug combinations using a hazards model with time-dependent covariates. *Biometrics*, **36**, 537.

STANISWALIS, J.G. & MCCRADY, C.W. (1988). The use of kernel estimators in describing human T lymphocyte proliferation induced by phorbol esters and $Ca^{2+}$ ionophore. *J. Am. Coll. Toxicol.*, **7**, 939.

TOBIAS, J.S., PARKER, L.M., TATTERSALL, M.H.N. & FREI, E. III (1975). Adriamycin/cyclophosphamide and adriamycin/melphalan in advanced L1210 leukaemia. *Br. J. Cancer*, **32**, 199.

WAMPLER, G.L., CARTER, W.H.Jr & WILLIAMS, V.R. (1978). Combination chemotherapy: arriving at optimal treatment levels by incorporating side effect constraints. *Cancer Treat. Rep.*, **62**, 333.

WILSON, J.D., CARTER, W.H.Jr, CAMPBELL, E.D., KESSLER, F.K. & CARCHMAN, R.A. (1986). Application of response-surface methodology to detect interactions of genotoxic agents in cultured mammalian cells. *J. Toxicol. Environ. Health*, **19**, 173.