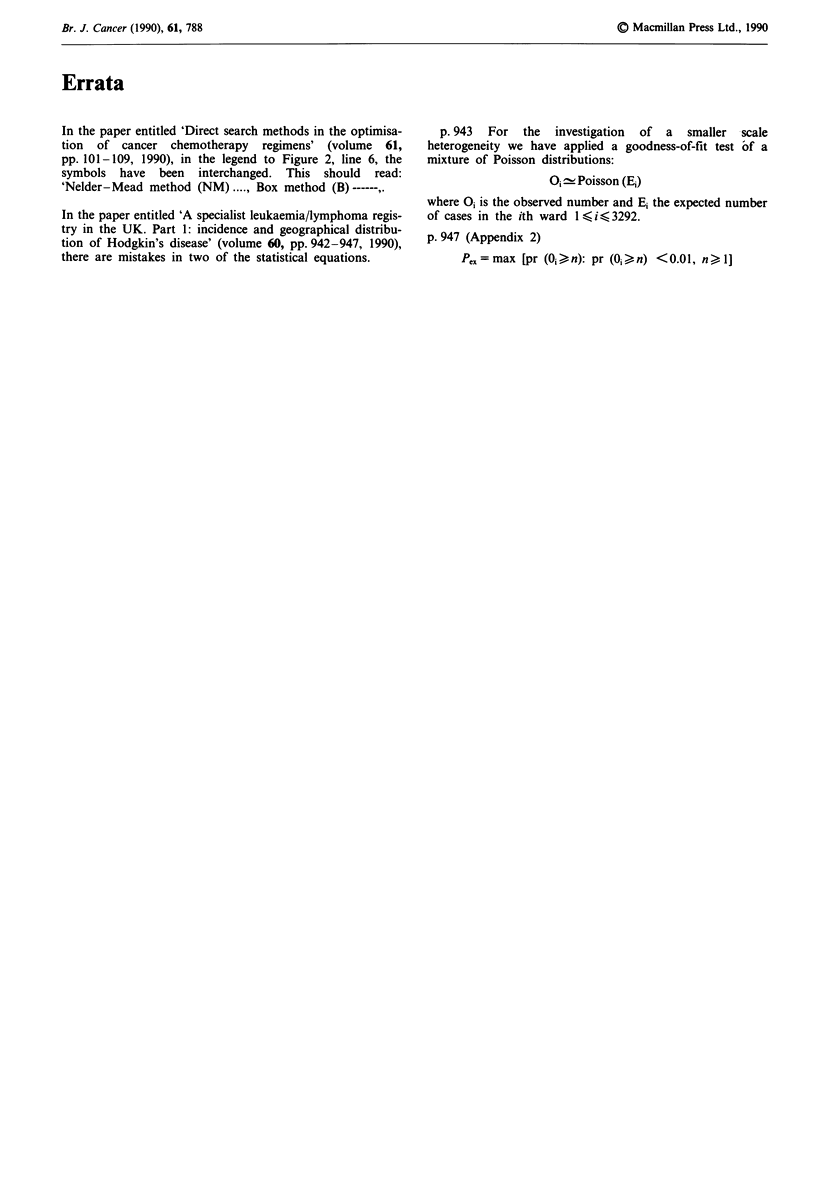# Direct search methods of cancer

**Published:** 1990-05

**Authors:** 


 © Macmillan Press Ltd., 1990

### Errata

In the paper entitled 'Direct search methods in the optimisation of cancer chemotherapy regimens' (volume **61**, pp. 101–109, 1990), in the legend to Figure 2, line 6, the symbols have been interchanged. This should read: 'Nelder–Mead method (NM) ...., Box method (B) ------,.